# Breast Organ Dose and Radiation Exposure Reduction in Full-Spine Radiography: A Phantom Model Using PCXMC

**DOI:** 10.3390/diagnostics15212787

**Published:** 2025-11-03

**Authors:** Manami Nemoto, Koichi Chida

**Affiliations:** 1 Course of Radiological Technology, Health Sciences, Tohoku University Graduate School of Medicine, 2-1 6 Seiryo, Aoba, Sendai 980-8575, Miyagi, Japan; manami.nemoto.r2@dc.tohoku.ac.jp; 2 Department of Radiation Disaster Medicine, International Research Institute of Disaster Science, Tohoku University, 468-1 Aramaki Aza-Aoba, Aoba, Sendai 980-0845, Miyagi, Japan

**Keywords:** breast cancer, scoliosis, cancer risk, full-spinal radiograph, organ dose, pediatric X-ray examination, radiation safety, radiation dose, Monte Carlo method, PCXMC

## Abstract

**Background/Objectives:** Full-spine radiography is frequently performed from childhood to adulthood, raising concerns about radiation-induced breast cancer risk. To assess such probabilistic risks as cancer, accurate estimation of equivalent and effective organ doses is essential. The purpose of this study is to investigate X-ray imaging conditions for radiation reduction based on breast organ dose and to evaluate the accuracy of simulation software for dose calculation. **Methods:** Breast organ doses from full-spine radiography were calculated using the Monte Carlo-based dose calculation software PCXMC. Breast organ doses were estimated under various technical conditions of full-spine radiography (tube voltage, distance, grid presence, and beam projection). Dose reduction methods were explored, and variations in dose and error due to phantom characteristics and photon history number were evaluated. **Results:** Among the X-ray conditions, the greatest radiation reduction effect was achieved by changing the imaging direction. Changing from the anteroposterior to posteroanterior direction reduced doses by approximately 76.7% to 89.1% (127.8–326.7 μGy) in children and 80.4% to 91.1% (411.3–911.1 μGy) in adults. In addition, the study highlighted how phantom characteristics and the number of photon histories influence estimated doses and calculation error, with approximately 2 × 10^6^ photon histories recommended to achieve a standard error ≤ 2%. **Conclusions:** Modifying radiographic conditions is effective for reducing breast radiation exposure in patients with scoliosis. Furthermore, to ensure the accuracy of dose calculation software, the number of photon histories must be adjusted under certain conditions and used while verifying the standard error. This study demonstrates how technical modifications, projection selection, and phantom characteristics influence breast radiation exposure, thereby supporting the need for patient-tailored imaging strategies that minimize radiation risk while maintaining diagnostic validity. The findings may be useful in informing radiographic protocols and the development of safer imaging guidelines for both pediatric and adult patients undergoing spinal examinations.

## 1. Introduction

Radiation is indispensable in the medical field; however, it is well-known that it carries risks [1,2,3]. Biological effects (stochastic and deterministic effects) may occur due to radiation exposure [4]. The radiation dose received during routine radiographic examinations is at a level that does not raise concern about the effects of radiation; however, in recent years, there have been reports of radiation damage due to an increase in the number of examinations and X-ray exposure time [5]. The International Commission on Radiological Protection and other organizations have urged caution in avoiding radiation related-health issues [6,7]. However, cases of radiation-induced skin and lens damage continue to be documented, highlighting ongoing concern [8,9,10,11].

Scoliosis typically develops during childhood, with spinal curvature and rotation progressing as growth continues [12,13,14,15,16,17,18,19]. Diagnosing and treating scoliosis requires visualizing the entire spine, which has traditionally been performed using full-spine X-ray imaging [20]. However, because these full-spine radiographic procedures are frequently performed over a long period from childhood to adulthood, an increased risk of breast cancer, a stochastic effect, has been reported in women since the 1980s [21,22,23,24]. This risk is particularly concerning in childhood, when sensitivity to X-rays is heightened [25,26,27,28,29]. To assess the risk associated with stochastic effects such as cancer, equivalent and effective doses to specific patient organs should be calculated [30].

Previous studies have reported varying radiation doses from full-spine radiography in scoliosis patients [31,32,33,34,35,36], but a key challenge remains: the relationship between specific radiographic protocols, such as tube voltage and the focus-image receptor distance (FID), and dose information during X-ray imaging is not clearly defined. In 2025, Japan updated its Diagnostic Reference Levels (DRLs), publishing the nation’s first DRL for pediatric full-spine radiography (specifically the surface air kerma value) [37]. However, the specific imaging methods remain at the discretion of each facility. In the late 2000s, the Ster EOS imaging system (EOS imaging) was developed for whole-body imaging with the goal of low-dose radiography. This dual-tube system enables rapid, high-quality imaging of the spine and lower limbs through simultaneous two-directional imaging [38]. However, its inability to perform supine imaging and its status as a large, dedicated device have hindered its widespread adoption in Japan [39,40,41]. Against this backdrop, Nemoto et al. proposed a method to reduce patient radiation exposure by determining the breast surface dose and organ dose under various X-ray conditions during conventional full-spine imaging [42]. This study was based on testing involving children (5 years old). Since body size changes with growth, the relationship between X-ray imaging conditions and radiation dose for larger body sizes remains unclear.

This study aimed to newly estimate breast organ doses in full-spine radiography for adults and to investigate X-ray imaging conditions (tube voltage, distance, grid presence, and imaging direction) for dose reduction based on breast organ doses in both pediatric and adult patients. Furthermore, the relationship between the number of photon histories, organ dose, standard error, and calculation time was determined in simulation software for dose calculation, and accuracy evaluation was performed. Based on these findings, modifying imaging conditions from conventional, widely adopted methods enables optimized radiation exposure tailored to individual patients and hospitals across a broader age range.

## 2. Materials and Methods

### 2.1. PCXMC-Monte Carlo Program

Breast organ doses can be estimated using PCXMC (a pc-based Monte Carlo program for calculating patient doses in medical x-ray examinations), a commercially available software developed by the Finnish Center for Radiation and Nuclear Safety, Säteilyturvakeskus. PCXMC calculates organ and effective doses in patients undergoing medical X-ray examinations. Common Monte Carlo calculation codes used in the medical field include MCNP5 (A General Monte Carlo N-Particle Transport Code [43]), EGS5 [44], and GEANT4 (Geometry and Tracking) [45], but these often require proficiency in computer operations and language development skills. PCXMC is a specialized computational software designed to evaluate patient radiation exposure in simple X-ray examinations, enabling easy dose assessments in clinical practice. Its convenience has led to widespread use for various purposes [46,47].

**Figure 1:**
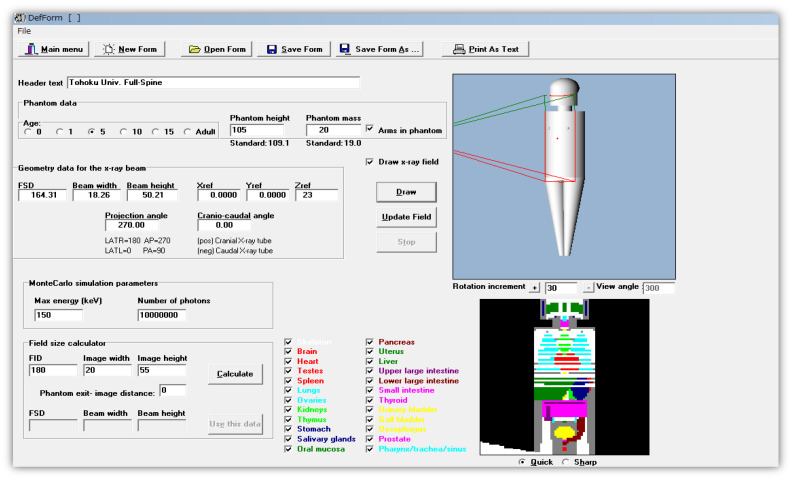
Input display for defining PCXMC examination conditions.

The PCXMC algorithm consists of three steps and estimates the absorbed dose in 29 biological organs and tissues. The steps are as follows: (A) defining examination conditions, (B) performing a Monte Carlo simulation, and (C) calculating the organ dose from the characteristic X-ray spectrum and patient incident dose. In step (A), to model the X-ray examination, the projection and range of the X-ray beam are defined at any position on the mathematical phantom displayed on the display, and the parameters necessary for calculation, such as FID and X-ray irradiation conditions, are entered. The phantom is based on the Cristy mathematical phantom model [48,49] and allows for the definition of arbitrary heights and weights for ages 0, 1, 5, 10, 15, and adulthood. In step (B), monochromatic energy photons are irradiated onto the human phantom set in (A), and the results of the Monte Carlo calculation of the energy accumulation in the organs are saved in a file. PCXMC performs calculations in 10 different batches for each energy value of the monochromatic photons, ranging from 10 to 150 kV. The estimated organ dose is represented by the average estimated dose for each energy value. The calculations include probabilistic uncertainty errors, and the standard error is estimated from the standard deviation of these batches [50]. At this stage, simulations are performed for a specified number of input photon histories per radiation field (NPhots). In the default settings of PCXMC, the NPhots is set to 2 × 10^4^, although it can be executed with any NPhots by changing the settings. However, setting a larger NPhots results in longer calculation times. According to Michael et al., in PCXMC’s simulation, assuming an NPhots 10^7^ can maintain the error hitting organs and tissues within the irradiation field at <1% [51]. In step (C), the X-ray spectrum is calculated using Birch & Marshall’s formula [52], and the organ dose calculated using photons of stored monochromatic energy is distributed according to the distribution ratio of continuous X-ray energy to calculate the organ dose. The PCXMC workflow is shown in Figure 1.

### 2.2. Calculation of Organ Dose to the Breast Using Full-Spine Imaging

PCXMC was used with version 2.0.1.2 installed on Windows XP Home Edition Version 2002. Inputting the conditions from Table 1 and NPhots 10^7^ into PCXMC, the breast organ doses of a child (5 years old) and an adult (30 years old) were calculated.

**Figure 2:**
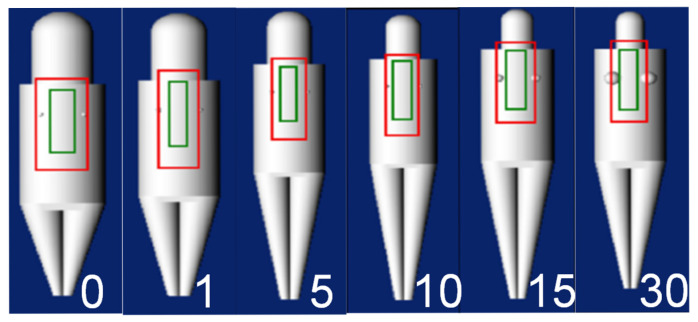
Age-specific PCXMC mathematical phantom.

**Figure 3:**
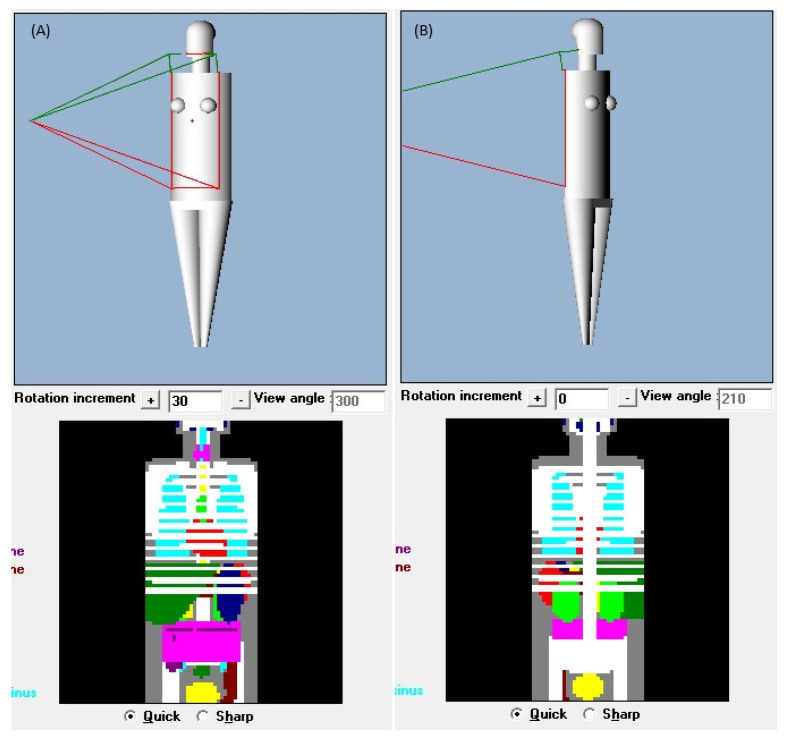
Irradiation field settings in the PCXMC adult phantom. (**A**) AP FID 180 cm. (**B**) PA FID 180 cm. AP, anteroposterior; PA, posteroanterior; FID, focus-image receptor distance.

The X-ray conditions assumed an imaging situation using a flat panel detector (FPD). Two patterns were assumed for the X-ray conditions: with and without the use of a scattered radiation removal grid. The scattered radiation removal grid (hereafter referred to as the grid) acts as a filter to remove the scattered rays generated when X-rays pass through a subject. Using the grid removes scattered radiation, resulting in higher-contrast images. However, it also absorbs some primary X-rays, necessitating an increase in the radiation dose to maintain image quality. For both pediatric and adult conditions, the mAs value was determined to maintain a consistent dose to the receptor of approximately 10–15 μGy without a grid and approximately 25–30 μGy with a grid. These doses were determined based on standard clinical protocols. Using this determined mAs value and a human phantom (whole-body phantom PBU-60, PBU-70: Kyoto Science Co., Ltd., Kyoto, Japan), the Entrance Air Kerma (EAK) was measured under the same X-ray conditions as those input into PCXMC and entered into PCXMC’s Input dose value. A compact semiconductor dosimeter, RaySafe X2 (RaySafe Ltd., Billdal, Sweden), was used to measure EAK. The height and weight of the PCXMC virtual phantom were changed from the initial settings to match the height and weight of the human phantoms (PBU-60, PBU-70) that are closer to the Japanese average. The 5-year-old phantom was set to 105 cm in height and 20 kg in weight, while the adult phantom was set to 165 cm in height and 50 kg in weight. Figure 2 shows examples of the displayed virtual phantoms by age. Four tube voltages (60, 80, 100, and 120 kV), two FIDs (120 and 180 cm), and two beam projections (anteroposterior [AP] and posteroanterior [PA]) were tested, resulting in 64 combinations. The long axis of the irradiation field was set to include the area from the lower edge of the orbit to the lumbar spine; if insufficient, the maximum long axis was used. To assess the dose to the breast, the short axis of the irradiation field was set to include the entire chest. Figure 3 shows examples of the irradiation field displays in the PCXMC adult phantom.

### 2.3. Relationship Among Breast Organ Dose, Standard Error, and Calculation Time Based on Changes in the Number of Photon History Sets

First, the conditions listed in Table 1 were entered into PCXMC. The number of photons was set to the initial settings of 2 × 10^4^ and 10^7^, which are considered to have a low error rate, as described above, and the breast organ dose and standard error were calculated for each setting. Assuming the calculation with 10^7^ photons as the true value, the mean absolute percentage error (MAPE) was calculated to investigate changes in the estimated organ dose. The MAPE calculates the absolute value of the difference between the predicted and true values for each data point, divides it by the true value, and outputs the average value by dividing the sum by the number of data points. It is defined as follows:

$$MAPE=\frac{100}{n}\sum_{i=1}^{n}|\frac{{\hat{y}}_{i}-y_{i}}{y_{i}}|$$

n: all data points;
$\hat{y}$_i_: predicted value;
$y_{i}$: true value.

In this study, the estimated organ dose calculated using NPhots 2 × 10^4^ was used as the predicted value, and that using NPhots 10^7^ was used as the true value. For each X-ray condition, the breast organ dose and standard error were compared based on differences in NPhots.

Next, the conditions in Table 2 were entered into PCXMC, and the relationship between NPhots, error, and calculation time was determined. Two phantom patterns (5-year-old child and 30-year-old adult) were selected, with a tube voltage of 80 kV, FID of 180 cm, two beam projections (AP and PA), two grid patterns (with and without), and five photon number settings (2 × 10^4^, 10^5^, 2 × 10^5^, 10^6^, 10^7^), resulting in a total of 20 patterns. The relationship between NPhots and the standard error was calculated and compared between adults and children. In addition, the calculation time required for each condition was measured, and the relationship between NPhots and the calculation time was determined. The time required to complete the calculations in step (B) was estimated.

## 3. Results

### 3.1. Calculation of Organ Dose to the Breast from Full-Spine Imaging

Table 3 and Table 4 present the estimated breast organ dose from full-spine imaging calculated using PCXMC under the X-ray conditions in Table 1 (NPhots 10^7^) and the standard error. Table 5 summarizes the average organ dose and adult-to-child ratio according to grid presence/absence and beam projection.

As shown in Table 5, the organ dose to the breast in children was approximately 17.0–40.1 µGy in the PA projection and approximately 86.4–366.8 µGy in the AP projection. The organ dose to the breast in adults was approximately 28.7–101.4 µGy in the PA projection and approximately 222.8–1000.0 µGy in the AP projection. Compared with those in pediatric patients, adult breast doses were 1.8–2.2 times higher without a grid and approximately 2.5–3.1 times higher with a grid.

**Figure 4:**
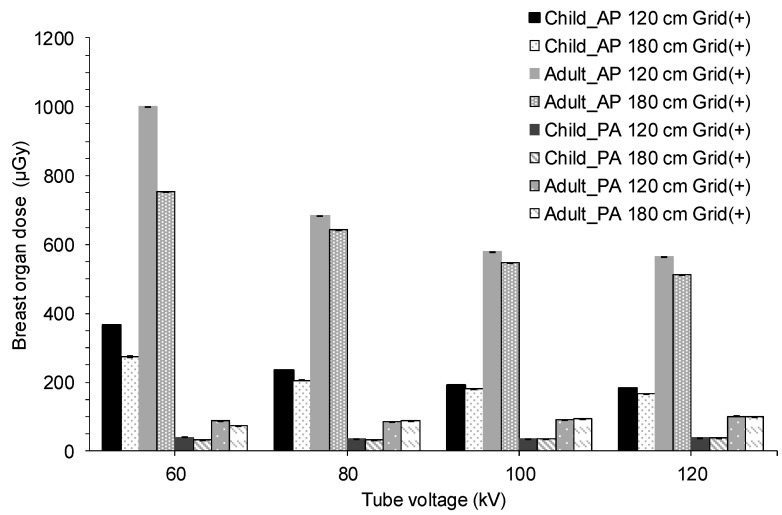
Breast organ dose for each X-ray condition calculated using PCXMC with a grid and 10^7^ photon histories. AP, anteroposterior; PA, posteroanterior.

**Figure 5:**
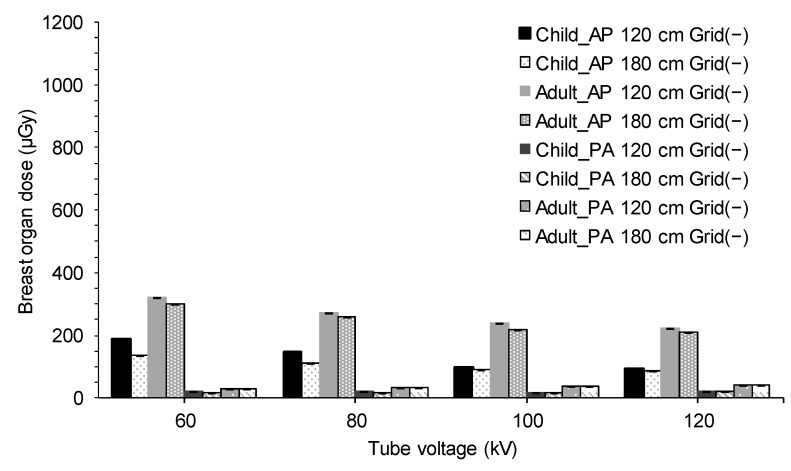
Breast organ dose for each X-ray condition calculated using PCXMC without a grid and 10^7^ photon histories. AP, anteroposterior; PA, posteroanterior.

The results shown in Table 3 and Table 4 are summarized in Figure 4 and Figure 5 for each X-ray condition, respectively. For both pediatric and adult AP radiographs, notable differences in organ doses were observed across different X-ray conditions. For AP imaging, lower tube voltage and shorter exposure distance resulted in higher breast doses.

### 3.2. Estimated Organ Dose to the Breast, Standard Error, and Calculation Time Based on Changes in the Number of Photon Sets

**Figure 6:**
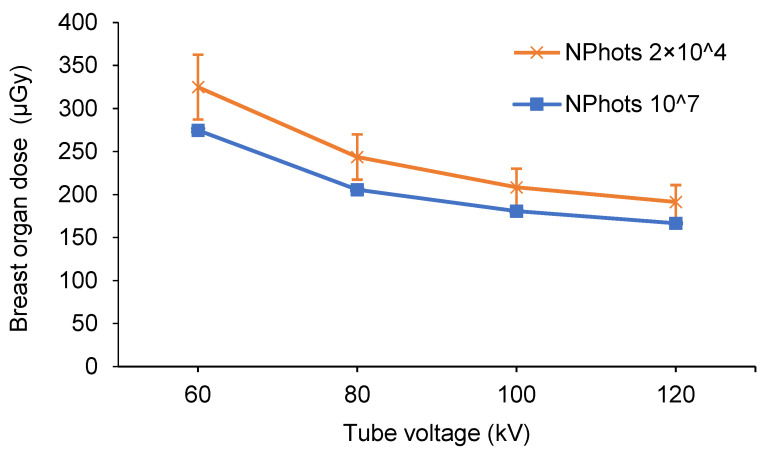
Differences in the breast organ dose and error due to the number of photon histories (pediatric phantom, AP, FID 180 cm, with grid). AP, anteroposterior; FID, focus-image receptor distance; NPhots, number of photon histories per radiation field.

**Figure 7:**
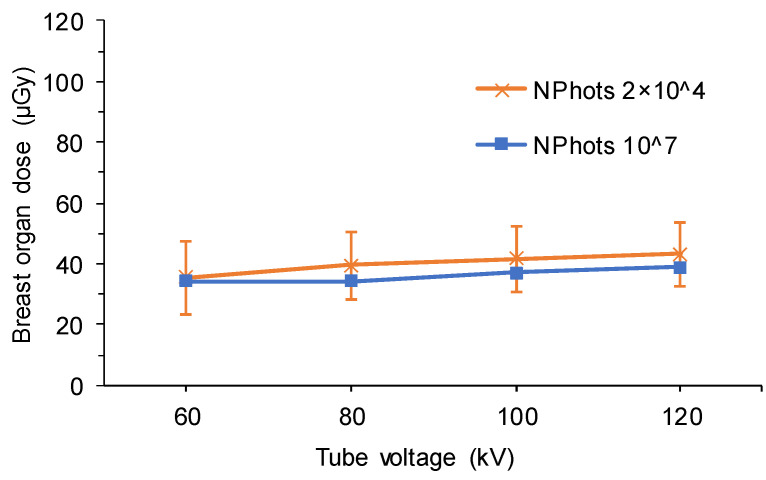
Differences in the breast organ dose and error due to the number of photon histories (pediatric phantom, PA, FID 180 cm, with grid). PA, posteroanterior; FID, focus-image receptor distance; NPhots, number of photon histories per radiation field.

**Figure 8:**
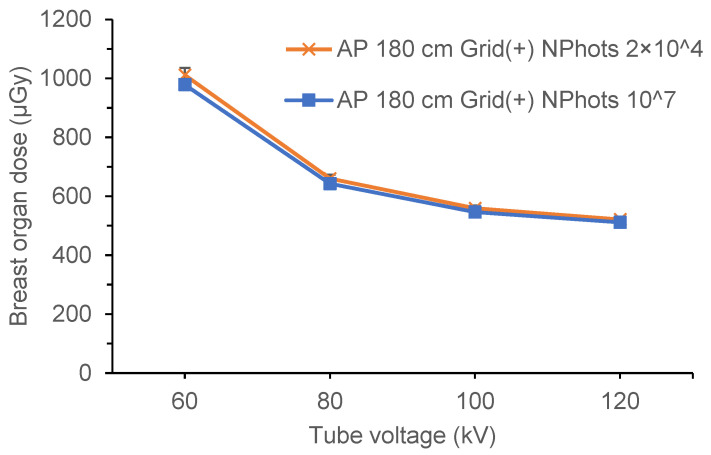
Differences in the breast organ dose and error due to the number of photon histories (adult phantom, AP, FID 180 cm, with grid). AP, anteroposterior; FID, focus-image receptor distance; NPhots, number of photon histories per radiation field.

**Figure 9:**
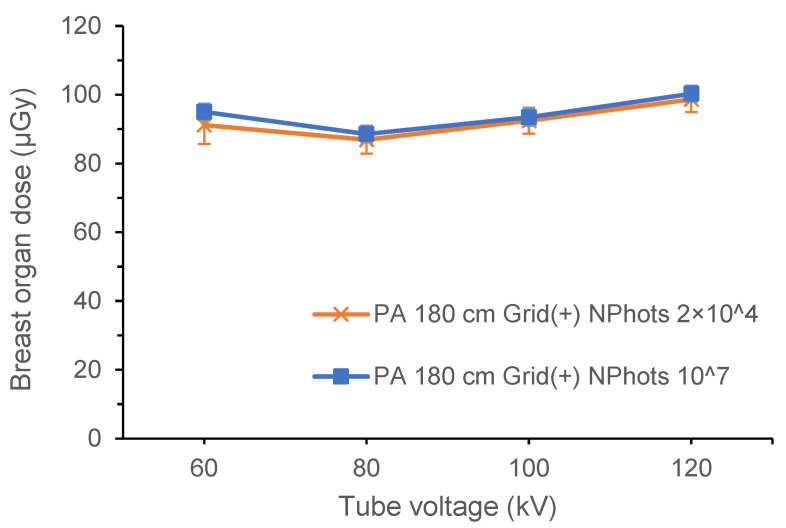
Differences in the breast organ dose and error due to the number of photon histories (adult phantom, PA, FID 180 cm, with grid). PA, posteroanterior; FID, focus-image receptor distance; NPhots, number of photon histories per radiation field.

Table 6 and Table 7 show the estimated organ dose to the breast and the standard error calculated for full-spine imaging under the X-ray conditions in Table 1 (NPhots 2 × 10^4^). Table 8 lists the average organ dose, average standard error, and MIDE for each condition. Comparisons of breast organ doses under pediatric AP, pediatric PA, adult AP, and adult PA conditions are shown in Figure 6, Figure 7, Figure 8 and Figure 9.

When the NPhots was changed, the difference in breast organ dose was greater in children, with the MAPE ranging from 10.6% to 16.7% in children and 2.1% to 2.6% in adults. When NPhots was 2 × 10^4^, the organ dose value was higher than when it was 10^7^ in children. The standard error increased as NPhots decreased in both the pediatric and adult phantoms. The standard error averaged 0.1–1.0% when NPhots was 10^7^ and 2.0–27.9% when NPhots was 2 × 10^4^. Additionally, the standard error was larger under PA projections than under AP projections in both children and adult phantoms.

**Figure 10:**
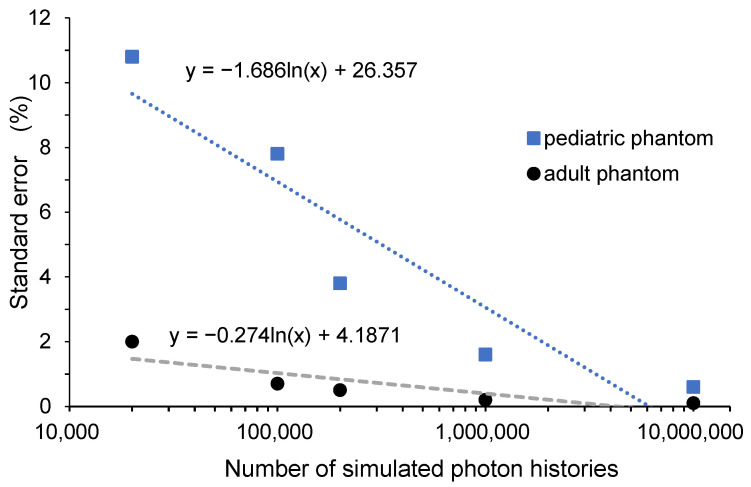
Relationship between the number of simulated photon histories and error in determining the breast organ dose from full-spine radiography.

**Figure 11:**
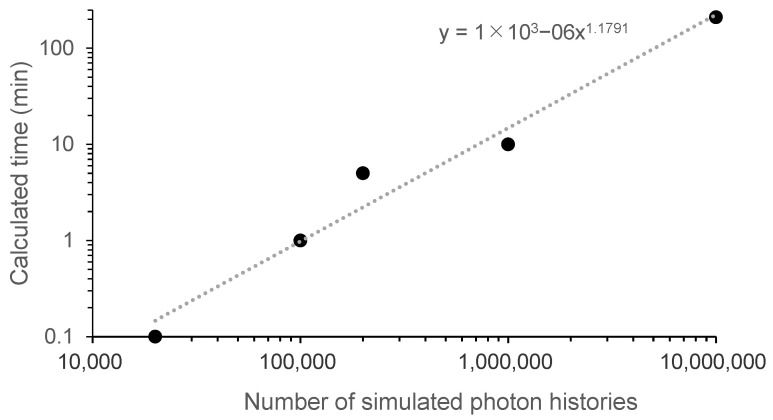
Relationship between the number of simulated photon histories and the calculated time in determining the breast organ dose from full-spine radiography (pediatric phantom, AP, FID 180 cm, with grid). AP, anteroposterior; FID, focus-image receptor distance.

The relationship between NPhots and standard error is shown in Figure 10. As NPhots increases, the standard error decreases, with a steeper reduction in children. The relationship between NPhots and the calculation time is shown in Figure 11. As NPhots increases, the calculation time also increases.

## 4. Discussion

### 4.1. Estimated Organ Dose to the Breast from Full-Spine Radiography

In orthopedics, when performing spinal radiography, it is common to use a grid in AP projections and lower the tube voltage compared to chest or abdominal radiography to improve contrast. However, these conditions tend to increase the breast cancer risk. Based on our findings, the most effective method for reducing radiation exposure in full-spine radiography is the selection of beam projection. Compared with AP projection, PA projection can reduce radiation exposure by 76.7–89.1% in children and approximately 80.4–91.1% in adults. This is because the PA projection prevents the breasts from being directly exposed to X-rays, and X-ray attenuation reduces the number of X-rays reaching the breasts. In actual imaging, the magnification factor of X-ray images varies slightly between AP and PA directions. This variation is not expected to affect Cobb angle measurements (the angle between vertebral bodies: an indicator of scoliosis progression), but details require verification through observer studies and phantom MTF/NEQ proxies. When performing PA radiography, markers must always be placed in a location not overlapping the vertebrae on the receptor, and the imaging method must be clearly communicated to the attending physician for follow-up. Furthermore, for patients with severe vertebral deformity who cannot assume a standing or prone position, performing PA radiography is difficult. In such cases, consider modifying the X-ray imaging conditions as follows.

The second most significant factor in reducing radiation exposure is the use of a grid. By examining the images without a grid, the number of X-rays required for an image can be reduced. Compared with using a grid, radiation exposure can be reduced by 36.6–50.2% in children and 59.2–67.8% in adults. Although the image sharpness may decrease without a grid, the image quality remains sufficient for measuring the Cobb angle, as mentioned earlier. Modern digital processing methods, such as virtual-grid processing and high-frequency enhancement processing, enable the acquisition of sharp images at low doses [53]. Additionally, the higher the tube voltage and imaging distance, the lower the organ dose. Specifically, changing the tube voltage from 60 kV to 80 kV can reduce radiation exposure to the breasts by approximately 19.5–36.3% in children and 14.3–34.3% in adults.

### 4.2. Estimated Organ Dose, Standard Error, and Computation Time for the Breast with Variations in NPhots

As shown in Table 8, the standard error increased when NPhots was small for both the pediatric and adult phantoms. This is because the estimated organ dose and its error depend on the number of interactions simulated within the organ. Generally, setting a larger photon number results in a smaller standard error. However, exceptions exist. According to the PCXMC User Guide, when the dose within an organ is low or the organ is small, the number of interactions may decrease even with a high photon number. This exception aligns with the finding that the standard error was large for PA imaging in both pediatric and adult cases under all X-ray conditions. In PA imaging, direct X-rays enter from the back, and the X-rays reaching the breast are reduced. Consequently, the dose within the breast of the virtual phantom is lower, leading to a decrease in the number of interactions. Therefore, it is important to note that the uncertainty in breast organ dose estimates for PA imaging is higher than for AP imaging.

Table 8 shows the differences in standard error between the pediatric and adult phantoms. The standard error was clearly higher in the pediatric phantoms when NPhots was low. As shown in Figure 10, the slope of the error with increasing NPhots was steeper for children than for adults. When NPhots was set to 10^7^, both groups exhibited similar standard errors. However, the difference in standard error became significant when NPhots was set to 2 × 10^4^. This suggests that differences in breast configuration within the phantom (e.g., the breast in the pediatric phantom is very small or coarsely defined) may reduce the number of interactions. When NPhots was varied, the MAPE for the pediatric breast organ dose increased. As mentioned above, this indicates a decrease in the accuracy of the estimated organ dose because the standard error is larger when NPhots is low.

Generally, in Monte Carlo methods, statistical error is used to quantify statistical variation to evaluate whether the number of interactions is sufficient. According to Curran et al., when considering the average dose, an error of 2% or less is considered clinically sufficient statistical power [54]. Figure 10 shows that to achieve an error of 2% or less, the required number of input photons is 2 × 10^4^ for adults and 2 × 10^6^ or more for children. Figure 11 indicates that the computation time per condition is a few seconds for adults but takes approximately 30 min for children. The computer used in this study was designed for general personal use; using a high-performance computer could potentially reduce computation time further.

### 4.3. Limitations

In this study, we performed calculations using only a 5-year-old pediatric phantom and an adult phantom. As discussed in Section 4.2., the estimated organ dose to the breast and its error may be influenced by the breast organ settings within the virtual phantom. Therefore, for the intermediate ages (10 years and 15 years), further investigation is needed to determine how the relationship between NPhots and standard error changes. Consequently, detailed recommendations regarding the optimal NPhots cannot be provided. Furthermore, regarding breast evaluation, the anatomical range and structure definitions for the breast in the PCXMC phantom are not publicly available. Therefore, the virtual phantom implemented within the PCXMC used in this study has limitations in its applicability. Follow-up studies using voxel phantoms for verification may yield more detailed information. Regarding the computation time, the recommended NPhots was presented for use on a typical home computer. However, because a further reduction in the computation time is expected when using a high-performance computer for calculations, entering NPhots higher than the recommended value whenever possible will yield estimates closer to the true values.

## 5. Conclusions

This study revealed that in full-spine radiography, PA imaging reduces breast radiation exposure by approximately 77–91% compared to AP imaging when X-ray conditions are tube voltage 60–120 kV and FID 120–180 cm. This finding was obtained through simulation using the dose calculation software PCXMC. To minimize statistical errors for both pediatric and adult cases, we recommend setting the minimum NPhots to 2 × 10^6^, with a goal error of ≤2%. Particular caution is required when using the software with pediatric phantoms as larger standard errors may lead to a slight overestimation of breast organ dose estimates. This study significantly contributes to the literature on radiation dose optimization by detailing how technical modifications, projection choices, and simulation software characteristics influence breast exposure. To minimize radiation exposure risk while evaluating the Cobb angle, we recommend verifying examinations using PA imaging at tube voltages ≥ 80 kV and no-grid, based on local qualification requirements. The findings may be useful in informing radiographic protocols and the development of safer imaging guidelines for both pediatric and adult patients undergoing spinal examinations.

## Figures and Tables

**Table 1:** X-ray conditions to input into PCXMC for calculating breast organ doses from full-spine radiography.

Projection	FID (cm)	Beam Width × Height at FID (cm^2^)		Tube Voltage (kV)	mAs				EAK (µGy)			
		Child	Adult		Child Grid (−)	Child Grid (+)	Child Grid (−)	Adult Grid (+)	Child Grid (−)	Child Grid (+)	Child Grid (−)	Adult Grid (+)
AP, PA	120	20 × 47	30 × 50	60	6.4	12.6	12.8	40.0	162.4	319.7	350.5	1090.0
				80	2.4	4.0	5.0	12.8	121.7	192.4	260.7	659.6
				100	1.0	1.9	2.6	6.3	78.4	150.8	211.5	514.2
				120	0.6	1.3	1.6	4.0	70.4	140.8	187.5	474.9
AP, PA	180	20 × 55	30 × 74	60	12.6	25.2	32.0	104.0	121.6	243.1	324.1	1052.5
				80	5.0	10.0	12.8	32.0	92.2	171.4	245.2	610.1
				100	2.6	5.0	6.3	16.0	73.8	144.3	191.4	477.0
				120	1.6	3.2	4.0	10.2	67.1	129.4	172.7	422.8

Full Spine, Filtration 2.8 mm Al, Additional filter 0 mm Cu, Anode angle 13, Oblique angle 0, NPhots 2 × 10^4^, 10^7^. AP, anteroposterior; PA, posteroanterior; FID, focus-image receptor distance; EAK, Entrance Air Kerma; NPhots, Number of photon histories per radiation field.

**Table 2:** X-ray conditions to input into PCXMC for determining the relationship between NPhots and error.

Projection	FID (cm)	Beam Width × Height at FID (cm^2^)		Tube Voltage (kV)	mAs		EAK (µGy)	
		Child	Adult		Child Grid (+)	Adult Grid (+)	Child Grid (+)	Adult Grid (+)
AP, PA	180	20 × 55	30 × 74	80	10.0	32.0	171.4	610.1

Full Spine, Filtration 2.8 mm Al, Additional filter 0 mm Cu, Anode angle 13, Oblique angle 0, NPhots 2 × 10^4^, 10^5^, 2 × 10^5^, 10^6^, 10^7^. AP, anteroposterior; PA, posteroanterior; FID, focus-image receptor distance; EAK, Entrance Air Kerma; NPhots, Number of photon histories per radiation field.

**Table 3:** Breast organ dose and error at 10^7^ photon histories in a pediatric phantom.

Projection	Tube Voltage (kV)	FID (cm)	Beam Width (cm, at FID)	Beam Height (cm, at FID)	Grid (−)			Grid (+)		
					mAs	Breasts (µGy)	Error (%)	mAs	Breasts (µGy)	Error (%)
AP	60	120	20	47	6.4	186.3	0.4	12.6	366.8	0.4
AP	80	120	20	47	2.4	147.9	0.4	4.0	233.8	0.4
AP	100	120	20	47	1.0	99.0	0.4	1.9	190.5	0.4
AP	120	120	20	47	0.6	91.3	0.4	1.3	182.7	0.4
AP	60	180	20	55	12.6	137.4	0.7	25.2	274.8	0.7
AP	80	180	20	55	5.0	110.6	0.6	10.0	205.6	0.6
AP	100	180	20	55	2.6	92.4	0.6	5.0	180.7	0.6
AP	120	180	20	55	1.6	86.4	0.6	3.2	166.6	0.6
PA	60	120	20	47	6.4	20.5	1.8	12.6	40.1	1.8
PA	80	120	20	47	2.4	22.2	1.5	4.0	35.0	1.5
PA	100	120	20	47	1.0	18.2	1.3	1.9	34.9	1.3
PA	120	120	20	47	0.6	19.5	1.2	1.3	38.6	1.2
PA	60	180	20	55	12.6	17.0	1.3	25.2	34.1	1.3
PA	80	180	20	55	5.0	18.5	1.1	10.0	34.2	1.1
PA	100	180	20	55	2.6	18.8	0.9	5.0	37.0	0.9
PA	120	180	20	55	1.6	20.1	0.8	3.2	38.9	0.8

Child Phantom, Full Spine, Filtration 2.8 mm Al, Additional filter 0 mm Cu, Anode angle 13, Oblique angle 0, NPhots 10^7.^ AP, anteroposterior; PA, posteroanterior; FID, focus-image receptor distance; NPhots, Number of photon histories per radiation field.

**Table 4:** Breast organ dose and error at 10^7^ photon histories in an adult phantom.

Projection	Tube Voltage (kV)	FID (cm)	Beam Width (cm, at FID)	Beam Height (cm, at FID)	Grid (−)			Grid (+)		
					mAs	Breasts (µGy)	Error (%)	mAs	Breasts (µGy)	Error (%)
AP	60	120	30	50	12.8	321.6	0.1	40.0	1000.0	0.1
AP	80	120	30	50	5.0	270.3	0.1	12.8	683.9	0.1
AP	100	120	30	50	2.6	238.0	0.1	6.3	578.7	0.1
AP	120	120	30	50	1.6	222.8	0.1	4.0	564.3	0.1
AP	60	180	30	74	32.0	301.3	0.1	104.0	978.6	0.1
AP	80	180	30	74	12.8	258.2	0.1	32.0	642.6	0.1
AP	100	180	30	74	6.3	219.1	0.1	16.0	546.1	0.1
AP	120	180	30	74	4.0	209.0	0.1	10.2	511.6	0.1
PA	60	120	30	50	12.8	28.7	0.3	40.0	88.9	0.3
PA	80	120	30	50	5.0	34.1	0.2	12.8	86.4	0.2
PA	100	120	30	50	2.6	37.1	0.1	6.3	91.2	0.1
PA	120	120	30	50	1.6	40.0	0.1	4.0	101.4	0.1
PA	60	180	30	74	32.0	29.3	0.3	104.0	95.0	0.3
PA	80	180	30	74	12.8	35.6	0.2	32.0	88.6	0.2
PA	100	180	30	74	6.3	37.5	0.2	16.0	93.4	0.2
PA	120	180	30	74	4.0	40.8	0.2	10.2	100.3	0.2

Adult Phantom, Full Spine, Filtration 2.8 mm Al, Additional filter 0 mm Cu, Anode angle 13, Oblique angle 0, NPhots 10^7^. AP, anteroposterior; PA, posteroanterior; FID, focus-image receptor distance; NPhots, Number of photon histories per radiation field.

**Table 5:** Comparison of average breast organ dose in full-spine radiography between children and adults.

	Average (µGy)			
	Grid (−) AP	Grid (−) PA	Grid (+) AP	Grid (+) PA
Child	118.9 (86.4–186.3)	19.4 (17.0–22.2)	225.2 (166.6–366.8)	36.6 (34.1–40.1)
Adult	263.2 (222.8–321.6)	35.4 (28.7–40.8)	688.2 (511.6–1000.0)	93.1 (86.4–101.4)
Adult/Child	2.2 (1.7–2.6)	1.8 (1.7–1.8)	3.1 (2.7–3.1)	2.5 (2.5–2.5)

Full Spine, Filtration 2.8 mm Al, Additional filter 0 mm Cu, Anode angle 13, Oblique angle 0, NPhots 10^7^. Average (min.–max.): breasts organ dose average (8 pattern; tube voltage 60, 80, 100, 120 kV, FID 120, 180 cm). AP, anteroposterior; PA, posteroanterior; FID, focus-image receptor distance.

**Table 6:** Breast organ dose and error at 2 × 10^4^ photon histories in a pediatric phantom.

Projection	Tube Voltage (kV)	FID (cm)	Beam Width (cm, at FID)	Beam Height (cm, at FID)	Grid (−)			Grid (+)		
					mAs	Breasts (µGy)	Error (%)	mAs	Breasts (µGy)	Error (%)
AP	60	120	20	47	6.4	192.8	12.9	12.6	380.1	12.9
AP	80	120	20	47	2.4	147.9	11.3	4.0	233.8	11.3
AP	100	120	20	47	1.0	99.0	10	1.9	190.4	10
AP	120	120	20	47	0.6	91.2	9.2	1.3	182.4	9.2
AP	60	180	20	55	12.6	162.5	11.6	25.2	325.0	11.6
AP	80	180	20	55	5.0	131.1	10.8	10.0	243.6	10.8
AP	100	180	20	55	2.6	106.6	10.4	5.0	208.4	10.4
AP	120	180	20	55	1.6	99.3	10.2	3.2	191.4	10.2
PA	60	120	20	47	6.4	22.6	31.5	12.6	44.3	31.5
PA	80	120	20	47	2.4	24.7	26	4.0	38.9	26
PA	100	120	20	47	1.0	19.1	22.7	1.9	36.6	22.7
PA	120	120	20	47	0.6	19.9	20.3	1.3	39.4	20.3
PA	60	180	20	55	12.6	17.7	33.6	25.2	35.4	33.6
PA	80	180	20	55	5.0	21.4	28	10.0	39.4	28
PA	100	180	20	55	2.6	21.2	25.8	5.0	41.6	25.8
PA	120	180	20	55	1.6	22.3	24	3.2	43.2	24

Child Phantom, Full Spine, Filtration 2.8 mm Al, Additional filter 0 mm Cu, Anode angle 13, Oblique angle 0, NPhots 2 × 10^4^. AP, anteroposterior; PA, posteroanterior; FID, focus-image receptor distance; NPhots, Number of photon histories per radiation field.

**Table 7:** Breast organ dose and error at 2 × 10^4^ photon histories in an adult phantom.

Projection	Tube Voltage (kV)	FID (cm)	Beam Width (cm, at FID)	Beam Height (cm, at FID)	Grid (−)			Grid (+)		
					mAs	Breasts (µGy)	Error (%)	mAs	Breasts (µGy)	Error (%)
AP	60	120	30	50	12.8	325.3	1.6	40.0	1007.7	1.6
AP	80	120	30	50	5.0	272.6	1.3	12.8	689.8	1.3
AP	100	120	30	50	2.6	239.8	1.1	6.3	590.1	1.1
AP	120	120	30	50	1.6	226.5	1	4.0	574.1	1
AP	60	180	30	74	32.0	312.0	2.4	104.0	1011.5	2.4
AP	80	180	30	74	12.8	265.5	2	32.0	659.9	2
AP	100	180	30	74	6.3	224.7	1.8	16.0	559.3	1.8
AP	120	180	30	74	4.0	212.2	1.7	10.2	521.3	1.7
PA	60	120	30	50	12.8	28.7	5.1	40.0	88.9	5.1
PA	80	120	30	50	5.0	34.7	4	12.8	87.9	4
PA	100	120	30	50	2.6	37.8	3.3	6.3	93.0	3.3
PA	120	120	30	50	1.6	41.0	3	4.0	103.8	3
PA	60	180	30	74	32.0	28.1	6	104.0	91.2	6
PA	80	180	30	74	12.8	35.0	4.7	32.0	86.9	4.7
PA	100	180	30	74	6.3	37.1	4.1	16.0	92.5	4.1
PA	120	180	30	74	4.0	40.2	3.7	10.2	98.6	3.7

Adult Phantom, Full Spine, Filtration 2.8 mm Al, Additional filter 0 mm Cu, Anode angle 13, Oblique angle 0, NPhots 2 × 10^4^. AP, anteroposterior; PA, posteroanterior; FID, focus-image receptor distance; NPhots, Number of photon histories per radiation field.

**Table 8:** Comparison of breast organ dose and average error based on differences in NPhots.

Phantom	Projection	NPhots	Average		MAPE (%)
			Breasts (µGy)	Error (%)	
Child	AP	10^7^	206.9	0.6	16.7
		2 × 10^4^	242.1	10.8	
	PA	10^7^	36.1	1.0	10.6
		2 × 10^4^	39.9	27.9	
Adult	AP	10^7^	613.2	0.1	2.6
		2 × 10^4^	629.7	2.0	
	PA	10^7^	88.8	0.2	2.1
		2 × 10^4^	87.0	4.6	

Full Spine, Filtration 2.8 mm Al, Additional filter 0 mm Cu, Anode angle 13, Oblique angle 0, NPhots 2 × 10^4^, 10^7^. 180 cm, Grid (+), Average (4 pattern; tube voltage 60, 80, 100, 120 kV). AP, anteroposterior; PA, posteroanterior; MAPE, mean absolute percentage error; NPhots, Number of photon histories per radiation field.

## Data Availability

The data presented in this study are available on request from the corresponding author due to privacy and ethical restrictions.

## Abbreviations

The following abbreviations are used in this manuscript:

NPhots	number of photon histories per radiation field
AP	anteroposterior
PA	posteroanterior
FID	focus-image receptor distance
MAPE	mean absolute percentage error
